# Rapid Quantification of Bacteria in Infected Root Canals Using Fluorescence Reagents and a Membrane Filter: A Pilot Study on Its Clinical Application to the Evaluation of the Outcomes of Endodontic Treatment

**DOI:** 10.1155/2012/172935

**Published:** 2012-05-27

**Authors:** Takuichi Sato, Keiko Yamaki, Naoko Ishida, Megumi Shoji, Emika Sato, Yuki Abiko, Kazuhiro Hashimoto, Yasuhisa Takeuchi, Junko Matsuyama, Hidetoshi Shimauchi, Nobuhiro Takahashi

**Affiliations:** ^1^Division of Oral Ecology and Biochemistry, Tohoku University Graduate School of Dentistry, Sendai 980-8575, Japan; ^2^Division of Periodontology and Endodontology, Tohoku University Graduate School of Dentistry, Sendai 980-8575, Japan; ^3^Division of Advanced Prosthetic Dentistry, Tohoku University Graduate School of Dentistry, Sendai 980-8575, Japan; ^4^Division of Pediatric Dentistry, Niigata University Graduate School of Medical and Dental Sciences, Niigata 951-8514, Japan

## Abstract

*Objective*. The bacterial examination has been performed during the course of the root canal treatment. In the present pilot study, the new developed method, using fluorescence reagents and a membrane filter, was applied to the detection and quantification of bacteria in infected root canals, in order to evaluate the outcomes of the treatment. *Methods*. Six infected root canals with periapical lesions from 5 subjects were included. Informed consent was obtained from all subjects (age ranges, 23–79 years). Samples from infected root canals were collected at the beginning of the treatment (termed #25 First), the end of the first day of treatment (termed #55 First), and the next appointment day (termed #55 Second). Then, the bacterial count (CFU) was measured using fluorescence reagents (4′,6′-diamidino-2-phenylindole and propidium iodide) and the polycarbonate membrane filter by Bioplorer. *Results*. The mean ± SD of CFU in the sample of “#25 First” was (1.0 ± 1.4) × 10^5^. As the root canal treatment progressed, the CFU decreased as 7.9 × 10^3^ (#55 First) and 4.3 × 10^2^ (#55 Second). *Conclusion*. In the present pilot study, rapid detection and quantification of bacteria in infected root canals were found to be successfully performed using fluorescence reagents and a membrane filter (Bioplorer analysis).

## 1. Introduction

It has been known that the microbiota in infected root canals is composed of various oral bacteria, in particular anaerobic bacteria [1–5], and is likely to lead to the failure of the treatment, such as the formation of persistent endodontic lesions. Thus, the bacterial examination during the course of the treatment of infected root canals has been performed in order to evaluate the outcomes of the treatment, resulting in good prognosis [6, 7]. However, detection of bacteria in infected root canal of each case by using culture and/or molecular biological techniques is relatively time consuming, and therefore these methods perhaps should be modified for the clinical use. Furthermore, it is still unclear whether the remaining small numbers of bacteria in the root canals have impact on the reoccurrence of periapical periodontitis or not.

 Recently, a rapid method of detection and quantification of bacteria in foodstuffs and/or chemical products, using fluorescence reagents and a membrane filter, has been developed in Japan [8, 9]. The technique enables us to count the number of live bacterial cells by differentiating live and dead bacterial cells in the sample within a half an hour. However, the method has not been applied to the quantification of samples in infected root canals.

 Therefore, in the present pilot study, we applied the new developed method using fluorescence reagents and a membrane filter to the detection and quantification of bacteria in infected root canals and compared its data with ones by culturing method.

## 2. Materials and Methods

### 2.1. Subjects

Subjects with periapical periodontitis (five females; age, 23–79 years), who were attending the Division of Endodontology, Tohoku University Hospital, were randomly selected for this study (Table 1). Periapical periodontitis was diagnosed based on clinical features, for example, sensitivity (tenderness) to percussion/occlusion and radiographical findings, as described previously [1]. Selected teeth had clinically no obvious margin leakages, had enough coronal structure for adequate isolation with a rubber dam, and were free of periodontal pockets deeper than 4 mm. Based on history, all subjects were medically healthy and received no antibiotics for the 3 months before sampling. Informed consent was obtained from all subjects, and this study was approved by the Research Ethics Committee of Tohoku University Graduate School of Dentistry, Sendai, Japan.

### 2.2. Sampling

Each tooth was isolated with a rubber dam, and the operative field was disinfected with both iodine glycerin dental disinfectants Showa (Showa Yakuhin Kako, Japan) and 70% ethanol. Coronal access cavity was prepared with a sterilized high-speed bur under irrigation with sterile saline solution. When the pulp chamber was exposed, a sterile #25 K-file (GC, Japan) was introduced, and the canal length was determined using an apex locator (Root ZX; Morita, Japan). Dentin sample (termed #25 First) was collected from an apical canal by filing intensively with a sterile K-file of the canal size. After the first sampling, cleaning and shaping of the root canal was carried out with sterile K-files (from #25 to #55) under alternative irrigation with 3% H_2_O_2_ and Antiformin JP Dental (Nihon Shika Yakuhin Co., Ltd., Japan), and dentin sample (termed #55 First) was again collected. Then, an intracanal medicament, that is, calcium hydroxide paste (UltraCal XS, Ultradent Products Inc., USA) was applied until the next appointment (for a week), and the coronal access cavity was sealed with a temporary cement (Lumicon; Heraeus Kulzer Japan, Japan).

 On the day of root canal obturation, each tooth was evaluated for clinical condition, and it was confirmed that there were no clinical signs of apical periodontitis as described above. The tooth was isolated with a rubber dam, and the operative field was disinfected as described above. The temporary cement was removed, and the intracanal medicament was rinsed out of the canal with sterile saline solution and a K-file. Immediately prior to root canal obturation with gutta-percha and sealer, dentin sample (termed #55 Second) was again collected. After obturation, the tooth was temporarily filled with glass ionomer cement (Fuji IX; GC). 

### 2.3. Quantification of Bacteria by Bioplorer

Each file cutoff by a sterilized wire cutter was placed in a sterile and endotoxin-free tube (BD, USA), and it was immediately transferred to a laboratory. Each sample was suspended in 4.0 mL of sterile, endotoxin-free saline (Otsuka Pharmaceutical Co., Ltd., Japan), and dispersed by vortexing for 1 min. The procedure of the quantification of bacteria by Bioplorer was described elsewhere [8, 9]. In brief, each 1.0 mL of sample was placed and fixed on a polycarbonate membrane filter (0.4 *μ*m pore size) (FJ-VKF03; Koyo Sangyo, Japan) by aspiration, after the addition of 100 *μ*L of Tween 80 (0.1%) (FJ-VKR05; Koyo Sangyo) to improve filterability. The membrane filter (triplicate) was stained with 100 *μ*L of 4′, 6′-diamidino-2-phenylindole (DAPI; 1.0 *μ*g/mL) (FJ-VKR01, Koyo Sangyo) (for live and dead bacterial cells due to its hydrophobic property and cell membrane permeability) for 2 min, and then stained with 100 *μ*L of propidium iodide (PI; 2.5 *μ*g/mL) (FJ-VKR03, Koyo Sangyo) (for only dead bacterial cells due to its ionic property and cell membrane impermeability). Subsequently, the number of live bacterial cells was monitored and calculated by Bioplorer (Koyo Sangyo), since the DAPI reacting with DNA produces luminescence (460 nm) by ultraviolet excitation light (375 nm), while the PI reacting with DNA does it (620 nm) by green excitation light (525 nm).

### 2.4. Quantification of Bacteria by Culture

The remaining 1.0 mL sample was dispersed with a Teflon homogenizer. Serial 10-fold dilutions (0.1 mL each) were spread onto the surface of CDC anaerobe 5% sheep blood agar (BD) plates (duplicate) and incubated in the anaerobic glove box (Hirasawa, Japan) at 37°C for 7 days, as described previously [1, 3, 4]. After the incubation, colony-forming units (CFUs) were counted.

### 2.5. Statistical Analysis

Wilcoxon test was used to determine the statistical significance of the bacterial counts determined by Bioplorer and culture, and two-way factorial ANOVA was used to determine the statistical significance of data of “#25 First,” “#55 First,” and “#55 Second.” A *P* value of <0.05 was considered to be statistically significant.

## 3. Results

**Figure 1:**
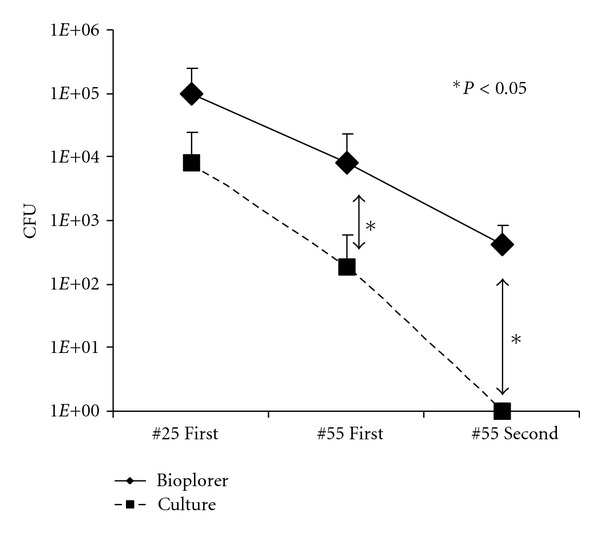
The mean bacterial counts (CFUs) in the samples (cases 1 to 6) at #25 First (at the beginning of the treatment), #55 First (at the end of the first day of treatment), and #55 Second (at the next appointment day) by Bioplorer and culture.

The mean ± SD bacterial counts (CFU) in the sample of “#25 First” were (1.0 ± 1.4) × 10^5^ and (0.83 ± 1.6) × 10^4^ by Bioplorer and culture, respectively (Figure 1). As the root canal treatment progressed, the CFU decreased as 7.9 × 10^3^ (#55 First) and 4.3 × 10^2^ (#55 Second) by Bioplorer analysis. Similarly, the CFU in the sample of “#55 First” and “#55 Second” analyzed by culture decreased compared with “#25 First,” and in particular, no bacteria were virtually detected in the sample of “#55 Second” by culture (Figure 1).

## 4. Discussion

Through the adoption of improved anaerobic glove box system, it has been reported that anaerobic bacteria are predominant in infected root canals, and thus suggesting that the environment of infected root canals may be anaerobic [1–5]. Therefore, the clinical examination of bacteria during the course of the treatment of infected root canals should be carefully performed, being concerned about anaerobic bacteria and anaerobic conditions. However, routine bacterial culturing procedure of root canal of each case, including incubation under anaerobic conditions, is relatively time consuming and seems somewhat clinically impractical.

 Alternatively, the detection and quantification of bacteria in infected root canals with molecular biological techniques has been applied in order to evaluate the outcomes of the treatment [10, 11]. Since the techniques require several hours to detect and quantify the bacteria (even though the methods are relatively quicker than culture), these methods perhaps should be modified for the clinical use. In the present pilot study, the new method, developed in Japan, using fluorescence reagents and a membrane filter, was applied to the detection and quantification of bacteria in infected root canals. The method made it possible to quantify the bacteria in the sample of infected root canals in approximately 20 min, indicating that the method is useful for the clinical bacterial examination during the course of the treatment of infected root canals when evaluating the outcomes of the treatment.

 Utilizing the Bioplorer, 1.0 × 10^5^, 7.9 × 10^3^ and 4.3 × 10^2^of bacteria were quantified from the samples of the beginning of the treatment (#25 First), the end of the first day of treatment (#55 First), and the next appointment day (#55 Second), respectively. A similar tendency of decreasing was observed in the quantification of bacteria by culture (Figure 1) in agreement with previous studies [12–18] although the counts by the Bioplorer were found to be higher than that by culture. The differences might be explained by the existence of unculturable bacteria in the infected root canals as well as in oral cavities [19–21].

 In all of the cases in the present study, obturation of root canals with gutta-percha and sealer were performed routinely after the evaluation for clinical conditions and confirmation with no clinical signs of apical periodontitis, as described previously [22] although several hundreds of bacteria were detected by Bioplorer (and no bacteria were virtually detected by culture). All of the cases in the present study showed good prognosis for 2–5 months after obturation completed (data not shown), suggesting that the small numbers of bacteria, even if they remained in the root canals, might have little impact on the reoccurrence of periapical periodontitis although further studies including long-term clinical observations are required to verify this proposition.

 In summary, in the present pilot study, focusing on the methodological confirmation regarding the accuracy and reliability when used clinical samples taken from infected root canals, detection, and quantification of bacteria in infected root canals were found to be performed rapidly using fluorescence reagents and a membrane filter (Bioplorer analysis).

## Figures and Tables

**Table 1:** Clinical features of subjects.

Case	1	2	3	4	5	6
Age	23	23	71	79	62	46
Gender	F	F	F	F	F	F
Tooth^a^	21	12	13	43	12	22

^a^A tooth of sampling site is expressed by the FDI two-digit notation.
